# A case of Marine Lenhart syndrome with Hashimoto’s thyroiditis that mimicked thyroid carcinoma

**DOI:** 10.1186/s12902-023-01438-8

**Published:** 2023-08-28

**Authors:** Peng Ye, Lan Li, Dong Wei, Fan Li, Yuan Zhong, Jing Zeng

**Affiliations:** 1 Department of Endocrinology and Metabolism, Obesity and Metabolic Diseases Care Center, The Second People’s Hospital of Chengdu, Chengdu, 610017 Sichuan China; 2 Medical Examination Center, The Second People’s Hospital of Chengdu, Chengdu, 610017 China; 3 Department of Pathology, The Second People’s Hospital of Chengdu, Chengdu, 610017 China

**Keywords:** Marine Lenhart syndrome, Hot thyroid nodule, Hashimoto’s thyroiditis, Case report

## Abstract

**Background:**

Marine Lenhart syndrome is a rare disease and causes refractory hyperthyroidism. So far, little evidence on the combination of both Marine Lenhart syndrome and Hashimoto's thyroiditis is available. We suspect that Marine Lenhart syndrome when combined with Hashimoto's thyroiditis might have its particular features, which are not exactly the same as those of the isolated Marine Lenhart syndrome.

**Case presentation:**

A 56-year-old middle-aged man presented with recurrent hyperthyroidism, and Graves' disease combined with Hashimoto's thyroiditis was considered. Radionuclide imaging showed a hot nodule, but ultrasonography suggested the possibility of malignancy with a category of 4B according to the Chinese-Thyroid Imaging-Reporting and Data System (C-TIRADS) model. Fine needle aspiration cytology (FNAC) revealed eosinophilic follicular lesions with papillary features, and prompted that papillary thyroid carcinoma could not be excluded. Partial thyroidectomy was performed and the nodule was proven to be benign by histopathology. The final diagnosis was atypical Marine Lenhart syndrome with Hashimoto's thyroiditis.

**Conclusions:**

Marine Lenhart syndrome combined with Hashimoto's thyroiditis has its particular characteristics, showing some signs mimicking malignancy. Limitations of ultrasonography and FNAC in diagnosis should be noted in these scenarios.

## Significance statement

Graves' disease with concomitant functional nodules, known as Marine Lenhart syndrome, is rare and causes refractory hyperthyroidism. Graves' disease combined with Hashimoto's thyroiditis is common in clinical practice, while evidence on Marine Lenhart syndrome combined with Hashimoto's thyroiditis is little. We report a case of atypical Marine Lenhart syndrome with Hashimoto’s thyroiditis presented with recurrent hyperthyroidism. The functional nodule of this patient showed some confusing signs mimicking thyroid carcinoma on both ultrasound and fine needle aspiration cytology (FNAC). We conclude that this might be the unique characteristic of the combination of both Marine Lenhart syndrome and Hashimoto’s thyroiditis. Limitations of ultrasonography and FNAC in diagnosis should be noted in the circumstances.

## Background

Graves' disease with concomitant functional nodules, known as Marine Lenhart syndrome, is rare and present in patients with refractory hyperthyroidism. According to previous data, the prevalence of Marine Lenhart syndrome in patients with Graves' disease is about 0.26–2.7% [1–4]. On radionuclide imaging, functional nodules appear as hot nodules. Graves' disease combined with Hashimoto's thyroiditis is common in clinical practice. Nodules in Hashimoto's thyroiditis show inhomogeneous patterns. Although it's rare, hot nodules (or areas) could still be seen in Hashimoto's thyroiditis [5]. So far, little evidence on the combination of both Marine Lenhart syndrome and Hashimoto's thyroiditis is available. We suspect that Marine Lenhart syndrome when combined with Hashimoto's thyroiditis might have its particular features, which are not exactly the same as those of the isolated Marine Lenhart syndrome previously reported.

## Case presentation

**Fig. 1:**
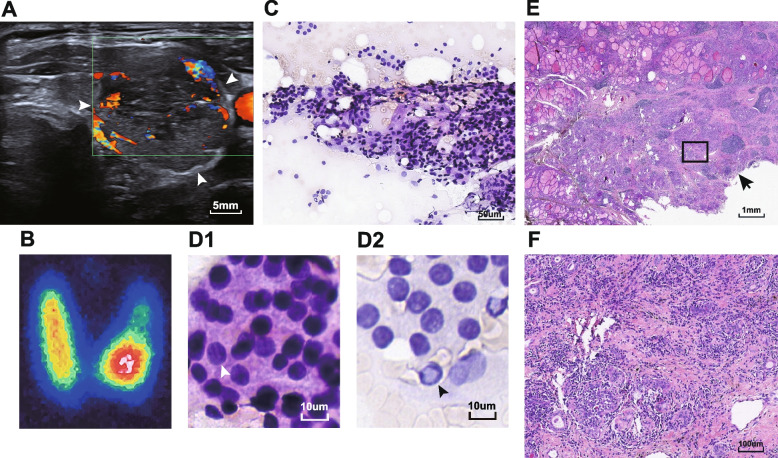
Imaging and pathology of the thyroid functional nodule. **A** Thyroid ultrasonography. The arrow reveals an inhomogeneous weak echogenic nodule in the left lobe of gland, about 21*16*29 mm in size, ill-defined margins, irregular shape. **B** Radionuclide imaging. A hot nodule in the left lobe of the gland corresponding to the weak echogenic nodule detected on ultrasound. **C** and **D** Fine needle aspiration cytology of the functional nodule (C*200-fold, D*600-fold). (D1) The arrow indicates nuclear grooves. (D2) The arrow indicates intranuclear pseudoinclusions. **E** and **F** Hematoxylin and eosin staining of the functional nodule (E*tenfold, F*100-fold). **E** The arrow indicates the ill-defined margins and irregular shape

A 56-year-old male patient was diagnosed as "hyperthyroidism" more than 6 years ago, and treated with methimazole for 2 years. Nearly 4 years ago, the patient had a recurrence of hyperthyroidism and was re-treated with methimazole. Half a month ago, without obvious inducement, the patient relapsed with heat intolerance, excessive sweating, palpitation and shortness of breath. He added the dose of methimazole to 10 mg twice daily by himself, and then presented to our hospital. On physical examination, his body temperature and heart rate were normal. A nodule was palpable in the left lobe of the thyroid without tenderness. Laboratory tests revealed a suppressed TSH at 0.010uIU/ml (normal range 0.35–4.94 uIU/ml), and a normal fT3 at 5.39 pmol/L (normal range 2.43–6.01 pmol/L), and a normal fT4 at 14.45 pmol/L(normal range 9.00–19.00 pmol/L). The TPOAb, TgAb and TRAb were all positive at > 1000 IU/ml (normal range 0.00–5.61 IU/ml), 6.60 IU/ml (normal range 0.00–4.11 IU/ml), and 6.94 IU/L (normal range 0.00–1.75 IU/L). ESR was mildly elevated at 36 mm/h (0–20 mm/h). Thyroid ultrasonography revealed an inhomogeneous weak echogenic nodule in the left lobe of gland, about 21*16*29 mm in size, ill-defined margins, irregular shape, and close relationship with shallow bread membrane (Fig. 1A). The nodule was classified into category 4B according to the Chinese-Thyroid Imaging-Reporting and Data System (C-TIRADS) model by the sonographer [6]. Moreover, enlarged left cervical lymph nodes were observed. (The patient provided a report of thyroid ultrasonography from another hospital one year before admission, and there was no such nodule). Radionuclide imaging of the gland demonstrated increased uptake of 99 m Tc pertechnetate and a hot nodule in the left lobe of the gland corresponding to the weak echogenic nodule detected on ultrasound (Fig. 1B). Based on the findings of ultrasound, and considering that the possibility of malignancy for hot nodules could not be completely excluded, we performed a FNA. Cytology revealed eosinophilic follicular lesions with papillary features, and papillary thyroid carcinoma cannot be excluded (Fig. 1C and D1, 2). Given all the evidence above, the patient was then transferred to the surgical department. Intraoperative frozen-section examination observed no definite neoplastic lesions. The type of surgical procedure was determined as left lobe and isthmus thyroidectomy plus central cervical lymph node dissection. Postoperative histopathology revealed autoimmune thyroiditis with granulomatous inflammation, interstitial fiber hyperplasia, and eosinophilic degeneration of some follicular epithelium with atypia, without definite evidence of malignancy (Fig. 1E and F). Immunophenotypes revealed TG( +), CK19(-), Galectin-3(-), HBME-1(-), and CD56( +). Coexisting with the decreased TSH, the functional nodule was considered TSH-independent. Thus, the patient was finally diagnosed as atypical Marine Lenhart syndrome with Hashimoto's thyroiditis. During follow up, thyroid function tests revealed euthyroidism at the first and the forth month after surgery, but a mildly suppressed TSH at 0.1020uIU/ml with an elevated TRAb at 2.20 IU/L, at the tenth month after surgery.

## Discussion

To the best of our knowledge, none has reported on the combination of both Marine Lenhart syndrome and Hashimoto's thyroiditis in a patient with recurrent hyperthyroidism this before. This case is unique, not just because of the presence of Marine Lenhart syndrome combined with Hashimoto's thyroiditis in patients with hyperthyroidism, but also because of the particular findings, which are not exactly the same as those in the isolated Marine Lenhart syndrome previously reported. In this case, the functional nodule has some confusing signs mimicking thyroid carcinoma on both ultrasound and FNAC. We conservatively dope out that ultrasonography and FNAC have certain limitations in differentiating benign and malignant nodules in cases of Graves' disease with Hashimoto's thyroiditis. On the other hand, the great significance of radionuclide imaging in recurrent hyperthyroidism with the coexistence of nodules, especially recently developed and rapidly growing ones, should be noticed. Previously, there were only two similar reports [7, 8]. One case was Marine Lenhart syndrome, which developed after thyroxine replacement therapy in a patient with Hashimoto's thyroiditis and hypothyroidism. Another case was Marine Lenhart syndrome in a newly diagnosed hyperthyroidism. In the latter case, diagnosis of Hashimoto's thyroiditis was not mentioned, whereas a markedly elevated TPOAb was observed. Both of the two cases had different pathogenic courses from that of our case, and did not provide complete pathological evidence (cytology or histopathology).

In the case we presented, production of hot nodules may be mostly related to the stimulation of TRAb, as discussed elsewhere [9, 10]. Due to coexistence of Hashimoto's thyroiditis, it may also be related to the various degrees of follicular degeneration and hyperplasia. Similarly, Hashimoto's thyroiditis, along with its focal or scattered infiltration of numerous lymphocytes and fibrosis of surrounding tissue, may contribute to a more confusing features on ultrasound, as inhomogeneous weak echo, ill-defined margins, irregular shape, etc. The most likely reasons for the potential misjudgment of FNAC might be the overlap of cytological characteristics. Actually, Hashimoto's thyroiditis has been reported as one of the most common lesions leading to false-positive diagnoses of FNAC [11]. At this point, another question excites our interest. It is whether Graves' disease when combined with Hashimoto's thyroiditis has a higher prevalence of Marine Lenhart syndrome as well as a faster growth of functional nodules. Further study are required to prove this.

Interestingly, this patient had a slightly elevated ESR, and the nodules showed findings, such as weak echogenic and ill-defined margins, suspected of subacute thyroiditis on ultrasound. Histopathology observed granulomatous inflammation. These signs suggest the possibility of subacute thyroiditis. However, based on the patient's medical history, symptoms, signs, and radionuclide imaging results, we think this is less likely. In clinical practice, subacute thyroiditis which occurs on a background of Hashimoto's thyroiditis, is difficult to be identified by auxiliary examination in some cases. Therefore, physicians should be aware of the importance of detailed history taking and physical examination, and not ignore the possibility of painless subacute thyroiditis. Nevertheless, multiple manifestations of functional nodules in Marine Lenhart syndrome combined with Hashimoto's thyroiditis are similar to subacute thyroiditis indeed.

Thyroid function tests showed a mildly decreased TSH along with an increased TRAb after surgery treatment. Accordingly, the trend of recurrent hyperthyroidism should be considered. This suggests that the type of surgical procedure might be inappropriate. In fact, given the risk of recurrence of hyperthyroidism, total or subtotal thyroidectomy are recommended by guidelines [12]. This case gives us some warning. Another crucial reminder is that the long-term prognosis of Marine Lenhart syndrome combined with Hashimoto's thyroiditis may be different from that of isolated Marine Lenhart syndrome. We must be alert to the risk of hypothyroidism and fully considered it when making treatment decisions. This also demonstrates the importance of a long-term follow up.

## Conclusions

Marine Lenhart syndrome should be considered in patients with refractory hyperthyroidism. Marine Lenhart syndrome combined with Hashimoto's thyroiditis has its particular characteristics, showing some signs mimicking malignancy. Limitations of ultrasonography and FNAC in diagnosis should be noted in these scenarios.

## Data Availability

The datasets generated during this study are available from the correspondences on reasonable request.

## Abbreviations

C-TIRADS: Chinese-Thyroid Imaging-Reporting and Data System
ESR: Erythrocyte Sedimentation Rate
FNAC: Fine Needle Aspiration cytology
fT3: Free Triiodothyronine
fT4: Free Tetraiodothyronine
TgAb: Thyroglobulin Antibody
TPOAb: Thyroid Peroxidase Antibody
TRAb: Thyrotropin Receptor Antibody
TSH: Thyroid Stimulating Hormone
